# Synergetic Theory of Information Entropy Based on Failure Approach Index for Stability Analysis of Surrounding Rock System

**DOI:** 10.3390/e25081237

**Published:** 2023-08-20

**Authors:** Lijun Xiong, Haiping Yuan, Gaoliang Liu, Hengzhe Li, Yangyao Zou, Xiaohu Liu, Xiaoming Li

**Affiliations:** 1 College of Civil and Hydraulic Engineering, Hefei University of Technology, Hefei 230009, China; xionglijun8@126.com (L.X.); gaoliangliu@163.com (G.L.); 2021010052@mail.hfut.edu.cn (H.L.); 18845761360@163.com (Y.Z.); zzlxm2012@126.com (X.L.); 2 College of Civil Engineering and Architecture, Anhui University of Science and Technology, Huainan 232001, China; 2020134@aust.edu.cn

**Keywords:** plastic shear strain, numerical limit analysis method, ultimate plastic shear strain, information entropy based on failure approach index, surrounding rock system

## Abstract

It is generally acknowledged that the stability evaluation of surrounding rock denotes nonlinear complex system engineering. In order to accurately and quantitatively assess the safety states of surrounding rock and provide a scientific basis for the prevention and control of surrounding rock stability, the analysis method of the synergetic theory of information entropy using the failure approach index has been proposed. By means of deriving the general relationship between the total two-dimensional plastic shear strain and the total three-dimensional plastic shear strain and obtaining the numerical limit analysis step of the plastic shear strain, the threshold value of the ultimate plastic shear strain can be determined, which has provided the key criterion for the calculation of the information entropy based on the failure approach index. In addition, combining with the synergetic theory of the principle of maximum information entropy, the evolution equation of the excavation step and information entropy based on the failure approach index of the surrounding rock system in underground mining space are established, and the equations of the general solution and particular solution as well as the expression of the destabilizing excavation step are given. To account for this, the method is applied to analyze the failure states of the floor surrounding rock after the mining of the 7_1_ coal seam in Xutuan Coal Mine and involve the disturbance effect and stability control method of the underlying 7_2_ coal seam roof from the macroscopic and microscopic aspects. Consequently, the validity of the analysis method of synergetic theory of information entropy based on the failure approach index has been verified, which presents an updated approach for the stability evaluation of surrounding rock systems that is of satisfactory capability and value in engineering applications.

## 1. Introduction

In recent years, subway tunnels, hydraulic tunnels, urban underground space, coal underground mining, and other projects have increased, and the stability of geotechnical bodies in underground engineering needs to be solved urgently. Therefore, the evaluation of the stability of the surrounding rock in underground engineering has been a research hotspot in the discipline of rock mechanics and engineering and a key technical difficulty that needs to be solved urgently in practical engineering [1,2,3,4].

In terms of the stability analysis of rock and soil mass, many scholars use various information entropy models to analyze and evaluate the stability of surrounding rock. Xu et al. [5] applied nonlinear scientific theories, such as the information entropy of plastic deformation, the mutation theory, etc., to investigate the nonlinear stability problems of rock mass so that the instability process and failure mechanism of rock mass could be revealed and a failure analysis method and instability criterion of rock mass that conform to reality could be deduced. According to the information entropy theory, Liu et al. [6] proposed a method for extracting the vibration characteristics of coal gangue based on the information entropy of the Hilbert spectrum and attained the outcome that the information entropy of the Hilbert spectrum when top coal falls performs better than that when mixing the coal gangue. In the light of the uncertainty measure theory, the information entropy theory, the reliability identification criteria, and the ranking criteria, He et al. [7] established a classification and ranking model of surrounding rock stability, providing a novel clue for the grading evaluation of tunnel surrounding rock stability. Li et al. [8] selected the formation lithology, the groundwater level, the rock occurrence, and the surrounding rock grade as risk evaluation indexes of water and mud inrush with respect to the geological and engineering factors of the tunnel, determined the index weights of each factor by virtue of the information entropy theory, and established an unascertained measurement model for risk evaluation of water and mud inrush. Zhao et al. [9] adopted the information entropy theory to determine the weight of each index and created a multi-index comprehensive measure evaluation model. Yang et al. [10] employed the stress information entropy principle to study the evolution law of mining-induced overburden stress, creating conditions for the establishment of the relationship between the change of stress information entropy and the evolution of mining-induced fractures, thereby laying a foundation for the formation of water channels and the identification of water hazard sources. In order to explore the damage evolution process of granite under triaxial compression, Ji et al. [11] introduced the AE amplitude information entropy variation coefficient and correlation dimension to identify granite failure precursors, presenting a fresh idea for rock instability prediction. With regard to the gray correlation analysis method and the entropy weight method, Zhang et al. [12] established a tunnel deformation analysis model for canopy settlement and lateral wall horizontal shrinkage during tunnel excavation and drew the conclusion that the main reason for the collapse accident of the Hilbert spectrum could be attributed to the tunnel overdigging. Moreover, Gao et al. [13] proposed a thermal infrared analysis method of exponential infrared image entropy to study the infrared radiation characteristics and fracture precursors during the process of rock fracture.

Zhang et al. [14] put forward the concept of failure approach index on the basis of yield approach index and quantitatively revealed the degree to which rock and soil mass approaches or reaches the yield or failure state in the most unfavorable way under loads. Hence, the calculation formula can be expressed as:

(1) $$\;\mathrm{FAI}=\{\begin{array}{cc} \omega & \left(0\leq \omega <1\right)\\ 1+\mathrm{FD} & \left(\omega =1,\mathrm{FD}\geq 0\right) \end{array},$$

where FAI represents the failure approach index; ω refers to the complementary parameter of yield approach index (YAI), ω=1−YAI; and FD signifies the failure degree:

(2) $$\mathrm{FD}=\gamma^{p}/\gamma_{f}^{p},$$

where $\gamma^{p}$ denotes the plastic shear strain; $\gamma^{p}=\sqrt{e_{ij}^{p}e_{ij}^{p}/2}$, and the plastic deviator strain $e_{ij}^{p}=\varepsilon_{ij}^{p}-\varepsilon_{m}^{p}\delta_{ij}$; and $\gamma_{f}^{p}$ stands for the ultimate plastic shear strain.

When 0≤FAI<1.0, the rock and soil mass is in the elastic stress state, and the greater the FAI value in this interval, the closer to the yield state it will become; when 1.0≤FAI<2.0, it is in the plastic yield state; at the time of FAI≥2.0, it is in the failure state.

**Figure 1:**
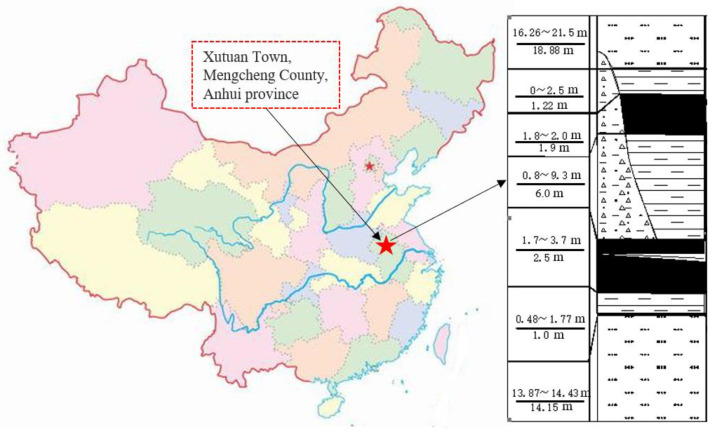
Geographical position of Xutuan Coal Mine and column diagram of coal and rock layers.

In simpler terms, the stability states of surrounding rock in engineering directly affect the stability control of surrounding rock. In the meantime, the plastic shear strain values and the ultimate plastic shear strain value of the surrounding rock are not prone to being quantitatively expressed through numerical calculation, and the surrounding rock has complicated efforts to determine the stability states. Accordingly, the analysis method of the synergetic theory of information entropy based on the failure approach index is introduced in this study to analyze this kind of problem. Under the condition of the principal strain order $\varepsilon_{1}\leq \varepsilon_{2}\leq \varepsilon_{3}$, the general relationship between the total two-dimensional plastic shear strain and the total three-dimensional plastic shear strain has been derived, and the numerical limit analysis method of plastic shear strain has been proposed so that the correctness and reliability of the method can be adequately validated. Therefore, the threshold of the ultimate plastic shear strain will be determined, which provides the essential criterion for the calculation of information entropy based on the failure approach index. In relation to the synergetic theory of the maximum information entropy principle, the evolution equation of the information entropy function based on the failure approach index and the excavation step in the surrounding rock system of underground mining space has been established. In addition, the general and solution formulae and the expression of the unstable excavation step are obtained, which provides a theoretical basis for the stability analysis of the surrounding rock system. Ultimately, the stability status of the 7_1_ coal seam floor in Xutuan Coal Mine (Figure 1) has been analyzed from the macroscopic and microscopic aspects. The research results will extend the failure approach index theory and present a method and technical reference for the stability evaluation of similar rock and soil masses.

## 2. Numerical Limit Analysis Method of Plastic Shear Strain

### 2.1. Relationship between Two-Dimensional Plastic Shear Strain and Three-Dimensional Plastic Shear Strain

In the numerical simulation software, the plastic shear strain adopts the three-dimensional plastic shear strain increment form. Nevertheless, the more concise two-dimensional plastic shear strain full form is often employed in the derivation and calculation of the theoretical model (for example, the full form $\gamma^{p}$ is used in the expression of the information entropy based on the failure approach index) [15]. In order to facilitate calculation, it is necessary to establish the relationship between the full form $\gamma^{p}$ and the incremental form $\Delta \varepsilon^{\mathrm{ps}}$.

Under the condition of the principal strain order $\varepsilon_{1}\leq \varepsilon_{2}\leq \varepsilon_{3}$, the expression of the full form $\gamma^{p}$ can be represented as:

(3) $$\;\;\gamma^{p}=\varepsilon_{3}^{\mathrm{ps}}-\varepsilon_{1}^{\mathrm{ps}},$$

where $\varepsilon_{3}^{\mathrm{ps}}$ and $\varepsilon_{1}^{\mathrm{ps}}$ signify the maximum and the minimum plastic principal strain, respectively.

For the purpose of facilitating the subsequent derivation, $\gamma^{p}$ is expressed in the incremental form:

(4) $$\;\;\Delta \gamma^{p}=\Delta \varepsilon_{3}^{\mathrm{ps}}-\Delta \varepsilon_{1}^{\mathrm{ps}}.$$

In the numerical simulation software, the strain-softened plasticity model adopts the incremental form $\Delta \varepsilon^{\mathrm{ps}}$, whose expression can be determined as:

(5) $$\Delta \varepsilon^{\mathrm{ps}}=\frac{\sqrt{{\left(\Delta \varepsilon_{1}^{\mathrm{ps}}-\Delta \varepsilon_{m}^{\mathrm{ps}}\right)}^{2}+{\left(\Delta \varepsilon_{m}^{\mathrm{ps}}\right)}^{2}+{\left(\Delta \varepsilon_{3}^{\mathrm{ps}}-\Delta \varepsilon_{m}^{\mathrm{ps}}\right)}^{2}}}{\sqrt{2}},$$

where

(6) $$\Delta \varepsilon_{m}^{\mathrm{ps}}=\left(\Delta \varepsilon_{1}^{\mathrm{ps}}+\Delta \varepsilon_{3}^{\mathrm{ps}}\right)/3.$$

Under the condition of the principal stress sequence $\sigma_{1}\leq \sigma_{2}\leq \sigma_{3}$, the expression of the shear plastic potential function $g^{s}$ [16] is obtained as:

(7) $$\;\;\;\;g^{s}=\sigma_{3}-\kappa \sigma_{1},$$

where

(8) $$\kappa =\frac{1-\sin\psi }{1+\sin\psi },$$

where ψ indicates the dilation angle.

With respect to the shear failure, the form of the non-associated flow rule is denoted as follows:

(9) $$\;\Delta \varepsilon_{i}^{\mathrm{ps}}=\lambda^{s}\frac{\partial g^{s}}{\partial \sigma_{i}}\left(i=1,3\right),$$

where $\lambda^{s}$ depicts the scaling factor.

According to Equation (9), the three plastic principal strain increment expressions can be attained as:

(10) $$\;\;\;\;\;\{\begin{array}{c} \Delta \varepsilon_{1}^{\mathrm{ps}}=\lambda^{s}\frac{\partial g^{s}}{\partial \sigma_{1}}=-\lambda^{s}\kappa \\ \Delta \varepsilon_{2}^{\mathrm{ps}}=\lambda^{s}\frac{\partial g^{s}}{\partial \sigma_{2}}=0\\ \Delta \varepsilon_{3}^{\mathrm{ps}}=\lambda^{s}\frac{\partial g^{s}}{\partial \sigma_{3}}=\lambda^{s} \end{array}.$$

In relation to Equation (10), the relationship between the principal strain increments $\Delta \varepsilon_{3}^{\mathrm{ps}}$ and $\Delta \varepsilon_{1}^{\mathrm{ps}}$ is established, and the expression can be signified as:

(11) $$\Delta \varepsilon_{1}^{\mathrm{ps}}=-\kappa \Delta \varepsilon_{3}^{\mathrm{ps}}.$$

In accordance with Equations (6) and (11), Equation (5) can be rewritten as:

(12) $$\Delta \varepsilon^{\mathrm{ps}}=\frac{\sqrt{3}}{3}\sqrt{1+\kappa +\kappa^{2}}\Delta \varepsilon_{3}^{\mathrm{ps}}.$$

Based on Equation (11), Equation (12) can be denoted as:

(13) $$\Delta \varepsilon^{\mathrm{ps}}=-\frac{\sqrt{3}}{3\kappa }\sqrt{1+\kappa +\kappa^{2}}\Delta \varepsilon_{1}^{\mathrm{ps}}.$$

Combined with Equations (4), (12) and (13), the incremental form $\Delta \varepsilon^{\mathrm{ps}}$ can be expressed as:

(14) $$\;\Delta \varepsilon^{\mathrm{ps}}=\frac{\sqrt{3}}{3}\sqrt{1+\kappa +\kappa^{2}}\frac{\Delta \gamma^{p}}{1+\kappa }.$$

With regard to Equation (14), $\Delta \varepsilon^{\mathrm{ps}}$ and $\Delta \gamma^{p}$ are affected by the dilation angle of rock and soil material ψ, and 0°≤ψ<90°. The ratio range of both can be determined as follows:

(15) $$\;\;\;\;\;\frac{1}{2}\leq \frac{\Delta \varepsilon^{\mathrm{ps}}}{\Delta \gamma^{p}}<\frac{\sqrt{3}}{3}.$$

Under the assumption of small deformation, no significant difference between the incremental form and the full form of plastic shear strain can be examined without considering the rotation of the stress spindle [17,18]. Therefore, the relationship between the full amount of the two-dimensional plastic shear strain $\gamma^{p}$ and the three-dimensional plastic shear strain $\varepsilon^{\mathrm{ps}}$ can be written as:

(16) $$\gamma^{p}\approx 2\varepsilon^{\mathrm{ps}}.$$

Under the limit strain state, Equation (16) can be represented as

(17) $$\;\;\gamma_{f}^{p}\approx 2\varepsilon_{f}^{ps},$$

where $\gamma_{f}^{p}$ and $\varepsilon_{f}^{ps}$ depict the two-dimensional and the three-dimensional ultimate plastic shear strain, respectively.

### 2.2. Calculation Process of the Ultimate Plastic Shear Strain

**Figure 2:**
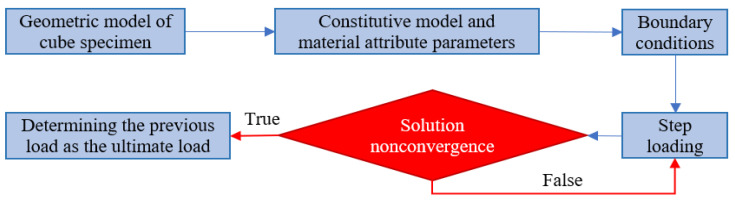
Numerical simulation flow chart of the ultimate load.

With the development of numerical methods for rock and soil mechanics, the numerical limit analysis method has gradually emerged, which has both comprehensive applicability and feasible practicability [19,20,21]. In this research, the ultimate load of rock and soil mass is determined by the overload method under the strain-softening constitutive model, which presents the prerequisite to defining the ultimate plastic shear strain of rock and soil mass, as displayed in Figure 2.

This study predominantly focuses on the ultimate plastic shear strain under the ideal plastic model and employs the numerical limit analysis method to determine the ultimate plastic shear strain of rock and soil materials. In the first place, with respect to the specific engineering background, the basic procedures, such as creating a geometric model, defining the strain softening constitutive model and parameters, setting boundary conditions, applying loads, etc., are established. In addition, the monitoring points for the plastic shear strain are set in the calculation model. Afterwards, the extraction and output programs of the plastic shear strain are self-compiled by the Python language and embedded in the numerical simulation program to output the plastic shear strain values of the monitoring points. Ultimately, the ‘axial load-plastic shear strain’ curve is drawn to compare and analyze the variation trend of the plastic shear strain at each monitoring point under each level of load and determine the ultimate plastic shear strain value of rock and soil materials.

**Figure 3:**
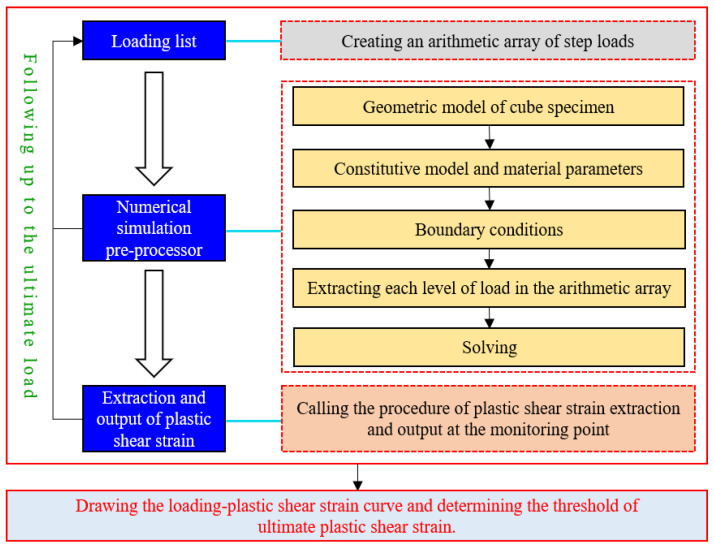
Numerical calculation flow chart of the ultimate plastic shear strain.

The key points of structural deformation weakness for the ultimate strain analysis of geotechnical materials have been normally selected. For instance, through the ultimate strain analysis of concrete blocks [22], 12 representative key nodes (elements) were chosen, and the load of each stage as well as the corresponding plastic shear strain values were extracted and output by means of the self-written Python program. Meanwhile, by comparing and analyzing the variation trends of the plastic shear strain of 12 key elements with the increase in axial load, the threshold value of the ultimate plastic shear strain of concrete material was finally determined. Therefore, the ultimate plastic shear strain in geotechnical engineering can also be obtained by the numerical limit analysis method, and the specific flow chart is depicted in Figure 3.

### 2.3. Solution and Verification of Ultimate Plastic Shear Strain

Given that there are numerous studies on the ultimate strain of concrete [23,24], the numerical limit analysis method is used to calculate and analyze the plastic shear strain of a C25 ordinary concrete cube specimen in this study. Hence, the ultimate plastic shear strain value of C25 ordinary concrete is obtained, and a comparison is made with the results of previous studies to verify the reliability of the numerical limit analysis method of plastic shear strain of C25 ordinary concrete.

**Figure 4:**
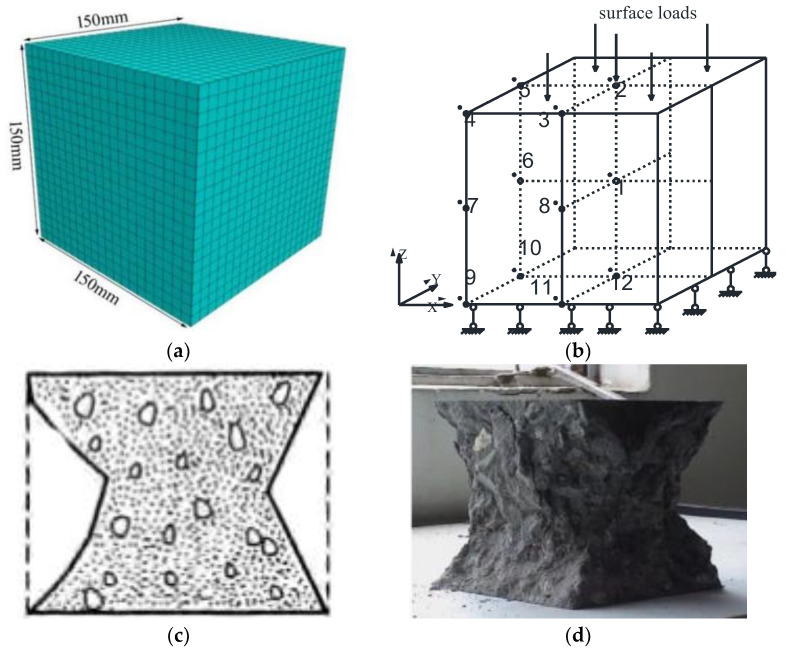
Calculation model: (**a**) geometric model; (**b**) boundary conditions and key nodes (units): key node (unit) 1~12 represent the monitoring node (unit) of plastic shear strain; (**c**) plane figure of failure morphology; and (**d**) real picture of failure morphology.

In this research, the finite difference software FLAC3D of version 6.0 is adopted to numerically simulate the C25 concrete cube specimen. First, the ultimate load of C25 concrete is determined by the overload method. The calculation model is a cubic specimen with a side length of 150 mm, and each side is divided into 20 grids, as shown in Figure 4a. Afterwards, the strain-softened plasticity model is employed, whose material attribute parameters are defined. Hence, the physical and mechanical parameters of C25 concrete are exhibited in Table 1. Furthermore, the bottom surface of the calculation model is fixed, while the top surface is subjected to a downward vertical plane load without considering the friction, as displayed in Figure 4b. According to the failure morphology of the cubic concrete specimen in Figure 4c,d, 12 key recording joints (elements) were set in the computational model (Figure 4b).

By means of the numerical simulation of the finite difference method, the main criteria to judge the failure include plastic zone penetration, the strength reduction method, the iteration non-convergence, etc. [26,27]. In this study, the failure criterion, whether the average force ratio can reach the default value of the convergence standard, is adopted. In the meantime, the axial load is imposed on the calculation model step by step until it achieves the failure state. Currently, the prior-level load refers to the ultimate load of the model.

**Figure 5:**
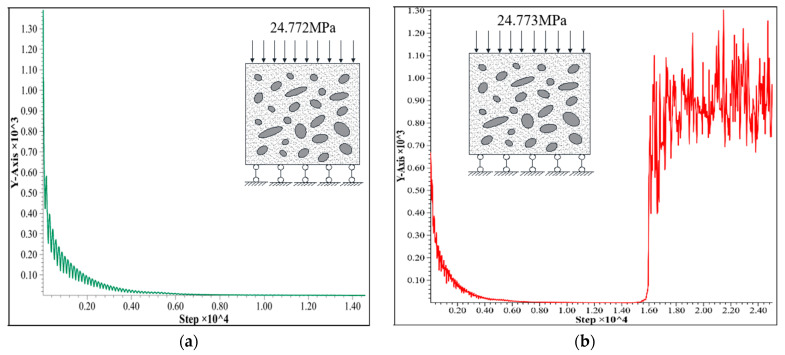
Curve of maximum unbalanced force: (**a**) area load on top surface with 24.772 MPa; (**b**) area load on top surface with 24.773 MPa.

During the process of numerical simulation, the maximum unbalance force of the C25 concrete cube specimen model was sampled and recorded. Figure 5a manifests the maximum unbalance force curve of 24.772 MPa for the C25 concrete cube specimen. In this case, there is a trend of an exponential decline and a rapid convergence in the curve of the middle section and rear section, which reveals that the model is apparently undamaged. Simultaneously, Figure 5b shows the maximum unbalance force curve that rises sharply at the last stage and keeps fluctuating up and down of 24.773 MPa for a C25 concrete cube specimen, which demonstrates that the calculation result displays non-convergence and the model is damaged. Therefore, the ultimate load of the specimen, 24.772 MPa, is basically in accordance with the strength of C25 concrete, which verifies the accuracy and reliability of the physical and mechanical parameters of C25 concrete.

In the finite difference software FLAC3D of version 6.0, the numerical limit analysis method is adopted to determine the ultimate plastic shear strain of C25 ordinary concrete. At that point, the basic steps can be presented as follows:

(1) Carry the load to the ultimate one by using the function ‘numpy.arange( )’ in the Python computer language and forming an arithmetic array;
(2) A cube specimen model with a size of 150 mm × 150 mm × 150 mm is constructed, whose sides are divided into 20 grids;
(3) Assign the strain-softened plasticity model and set the corresponding material attribute parameters (including softening parameters);
(4) Apply the displacement limits in all directions at the bottom of the model and impose the uniform load at the top side;
(5) Extract the load from the arithmetic array and solve it to the equilibrium state;
(6) According to the failure morphology of an ordinary concrete cube specimen, 12 key nodes (units) are set in the calculation model as monitoring points of plastic shear strain. Meanwhile, the coordinates of key nodes are loaded with the function ‘numpy.array( )’ in the Python computer language. In addition, the function ‘griddata( )’ is used to extract the plastic shear strain values of 12 key nodes under each level of load. Finally, the function ‘open( )’ is adopted to output the values of each level of load and the plastic shear strain values of 12 key nodes;
(7) Repeat the steps (1–6) and cycle to the end of the ultimate load;
(8) Draw the ‘axial load-plastic shear strain’ curve of 12 key nodes (elements). Under the function of the ultimate load, the maximum value of the plastic shear strain occurs at the upper part or corners of the model [22], and the damage to the upper side elements will inevitably lead to the overall failure of the specimen model. Therefore, the maximum value of the plastic shear strain is taken as the ultimate plastic shear strain of geotechnical materials.

**Figure 6:**
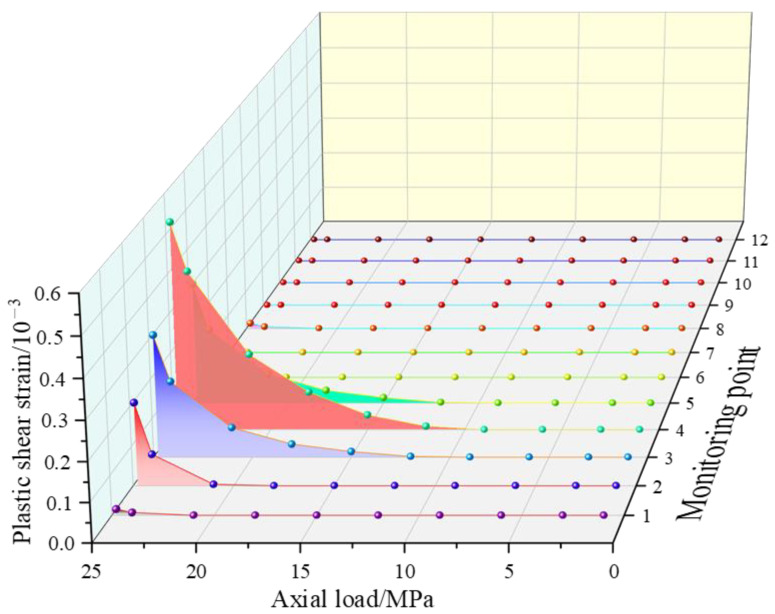
Relational graph between the axial load and the plastic shear strain.

Through the aforementioned basic steps, the relationship curve between the axial load and the plastic shear strain of 12 key elements in the C25 ordinary concrete cube specimen model is depicted in Figure 6. Among them, the plastic shear strains of units 9–12 become extremely small, and the plastic shear strain of unit 4 takes up the largest proportion, and failure occurs. Since unit 4 is located on the upper side of the calculation model, the damage to the element will inevitably contribute to the failure of the entire model. Thus, the plastic shear strain of this element signifies the ultimate plastic shear strain, which equals 0.53‰. With respect to Equation (17), the two-dimensional ultimate plastic shear strain $\gamma_{f}^{p}$ of an ordinary C25 concrete specimen is equivalent to 1.06‰.

Under the condition of the principal strain sequence $\varepsilon_{1}\leq \varepsilon_{2}\leq \varepsilon_{3}$, the numerical limit analysis method of plastic shear strain is employed to obtain the ultimate plastic shear strain value of C25 common concrete as 1.06‰. In addition, the ultimate plastic shear strain value of C25 common concrete, as given in the literature [22], equals 1.08‰. Thus, the results of these two studies remain approximately identical, which has verified the reliability of the numerical limit analysis method of plastic shear strain.

## 3. Synergetic Theory of Information Entropy Based on the Failure Approach Index

### 3.1. Definition of the Information Entropy Based on the Failure Approach Index

Assuming that the rock mass engineering system is equipped with *n* elements of given topological forms, boundary conditions, and applied loads, the failure approach index of the *i*-th element is $q_{i}$. Hence, the total failure approach index of the rock mass engineering system Q can be expressed as:

(18) $$Q=\sum_{i=1}^{n}q_{i},$$

constructs:

(19) $$\;\lambda_{i}=q_{i}/Q,$$

Therefore, there are:

$$\;\;\;\sum_{i=1}^{n}\lambda_{i}=1,\;\lambda_{i}\geq 0\;\;\;\;\;\;\left(i=1,2,\cdots ,n\right).$$

Remarkably, the mechanical meaning of the complete and non-negative physical quantity $\lambda_{i}$ represents the proportion of the failure approach index of the *i*-th element in the total one; that is, the distribution of the failure approach index in the rock mass engineering system can be described by $\lambda_{i}$. In order to comprehensively reflect the disorder indicated by the local state variables of the whole system, the information entropy function based on failure approach index s of the rock mass engineering system is defined as:

(20) $$s=-\phi \sum_{i=1}^{n}\lambda_{i}ln\left(\lambda_{i}\right),$$

where ϕ symbolizes a positive constant and normally equals 1.

### 3.2. Evolution Equation of Synergetic Theory of Information Entropy Based on Failure Approach Index

In order to facilitate the calculation, two variables (s,x) are selected in this study to describe the stope evolution process of the surrounding rock system in underground mining space. In accordance with the synergetic theory, the evolution equation of the system can generally be written in the form of the Langevin equation [28]:

(21) $$\dot{s}=K\left(s,x\right)+F\left(l\right),$$

where s signifies the slow variable, x denotes the fast variable, and K(s,x) depicts a nonlinear function containing the slow variable and the fast variable; l represents the excavation step, and F(l) refers to the fluctuation force.

Equation (21) demonstrates that the evolution process of any nonlinear system is controlled by the internal factors of the system, which can be expressed by the nonlinear function K(s,x). On the other hand, it is also affected by external random factors, which can be replaced by the fluctuation force F(l).

Indeed, system evolution lies at the root of the internal factors, whereas the role of external factors is mainly manifested in promoting variations in the internal factors and triggering them at critical points of qualitative change. To be more specific, the fluctuation force F(l) belongs to an external factor that has no decisive effect on the stability of the surrounding rock system in underground mining space, so the fluctuation term F(l) in Equation (21) can be ignored. Regarding the two-dimensional systems, the nonlinear function in Equation (21) can be represented as:

(22) K(s,x)=as−sx.

According to Equations (21) and (22), it can be realized as:

(23) $$\;\;\;\{\begin{array}{c} \dot{s}=as-sx\\ \dot{x}=-\beta x+s^{2} \end{array}.$$

By eliminating the fast variable x from Equation (23), the following integral expression can be obtained:

(24) $$x\left(l\right)=\int_{-\infty }^{l}e^{-\beta \left(l-\tau \right)}s^{2}\left(\tau \right)d\tau .$$

The integration by parts method can transform x(l) in Equation (24) into a function of s(l), namely:

(25) $$x\left(l\right)=\frac{1}{\beta }s^{2}(l)-\frac{1}{\beta }\int_{-\infty }^{l}e^{-\beta \left(l-\tau \right)}2{\left(s\dot{s}\right)}_{\tau }d\tau ,$$

when s changes slowly, it can be treated as a small quantity. By ignoring the integral term in Equation (25), we can obtain:

(26) $$x\left(l\right)\approx bs^{2}l,$$

where β signifies the growth rate of a fast variable, b=1/β.

By substituting Equation (26) into Equation (23), the evolution equation of information entropy based on failure approach index and stope excavation step of surrounding rock systems in underground mining space can be attained, namely:

(27) $$\frac{ds}{dl}=as-bs^{3}.$$

The general solution of Equation (27) can be derived as follows:

(28) $$s\left(l\right)=\sqrt{\frac{ae^{2al+2a\Delta C}}{1+be^{2al+2a\Delta C}}},$$

where a and b represent the undetermined coefficients, C indicates any constant, and l depicts the excavation step.

Since the influence of the fluctuation term *F*(*l*) is ignored, there are some errors in Equation (27). In order to reduce the effect of such errors, the gray system method is employed to accumulatively process the original sequence data of information entropy based on the failure approach index and dilute the impact of random factors (the fluctuation terms) on the original sequence data of information entropy [29]. Let s be the non-negative sequence of the original information entropy based on the failure approach index, and the new sequence generated after one-time accumulation refers to $s^{\prime }$, that is:

(29)  s={s(1),s(2),⋯,s(n)},

(30) $$s^{\prime }=\left\{s^{\prime }\left(1\right),s^{\prime }\left(2\right),\cdots ,s^{\prime }\left(n\right)\right\},$$

where *n* denotes the number of excavation steps.

The expression for the new sequence generated by a single summation is:

(31) $$s^{\prime }\left(i\right)=s^{\prime }\left(i-1\right)+s\left(i\right).$$

New sequence data created by mean values can be calculated as follows:

(32) $$\;Z\left(i\right)=[s^{\prime }\left(i-1\right)+s^{\prime }\left(i\right)]/2.$$

Hence, Equation (27) can be written as:

(33) $$\frac{ds^{\prime }}{dl}=as^{\prime }-b{\left(s^{\prime }\right)}^{3}.$$

The values of a and b in Equation (33) can be obtained by using the least squares estimation method:

(34) $$\left[\begin{array}{c} a\\ b \end{array}\right]={\left(B^{T}B\right)}^{-1}B^{T}E,$$

where:

(35) $$B=\left[\begin{array}{c} \begin{array}{cc} Z\left(2\right) & -Z^{3}\left(2\right) \end{array}\\ \begin{array}{cc} Z\left(3\right) & -Z^{3}\left(3\right) \end{array}\\ \begin{array}{cc} \vdots \;\;\; & \;\;\;\;\;\;\;\;\;\;\;\vdots \end{array}\\ \begin{array}{cc} Z\left(n\right) & -Z^{3}\left(n\right) \end{array} \end{array}\right],$$

(36) $$E={\left[s\left(2\right),s\left(3\right),\cdots ,s\left(n\right)\right]}^{T}.$$

The specific solution of Equation (33) can be attained as follows:

(37) $$s^{\prime }\left(l\right)=\sqrt{\frac{a}{\frac{e^{2al_{1}}}{\left[\frac{{\left(s^{\prime }\left(1\right)\right)}^{2}}{a-b\times {\left(s^{\prime }\left(1\right)\right)}^{2}}\right]e^{2al}}+b}}.$$

In relation to Equation (33), the right-side term actually represents the change rate of information entropy. Therefore, the excavation step corresponding to the point of maximum change rate is taken as the unstable excavation step of the surrounding rock system in underground mining space, and construction:

(38) $$P=as^{\prime }-b{\left(s^{\prime }\right)}^{3}.$$

When $\frac{dP}{ds^{\prime }}=0$, at this point, P can get a maximum value, and the information entropy corresponding to its maximum value is:

(39) $$s^{\prime }\left(l\right)=\sqrt{a/\left(3b\right)}.$$

By combining Equations (37) and (39), the excavation step corresponding to the instability of the surrounding rock system in underground mining space can be acquired, namely:

(40) $$l^{\prime }=\frac{1}{2a}ln\left(\frac{a-b\times {\left(s^{\prime }\left(1\right)\right)}^{2}}{2b\times {\left(s^{\prime }\left(1\right)\right)}^{2}}\right)+l_{1},$$

where $l_{1}$ represents the initial sequence number of excavation steps, which generally equals one and stays constant.

The expression of the safety factor of the surrounding rock system in underground mining space can be expressed as follows:

(41) $$\;\eta =l^{\prime }/L,$$

where η signifies the safety factor; $l^{\prime }$ denotes the unstable excavation step in the synergetic theory of information entropy based on the failure approach index; and L symbolizes the actual excavation step of the problem. If η>1, the surrounding rock system of underground mining space is in a stable state; if η≤1, it is in an unstable state.

## 4. Stability Analysis of the Stope Floor Surrounding Rock System in a Bifurcated Coal Seam

### 4.1. Project Overview

**Figure 7:**
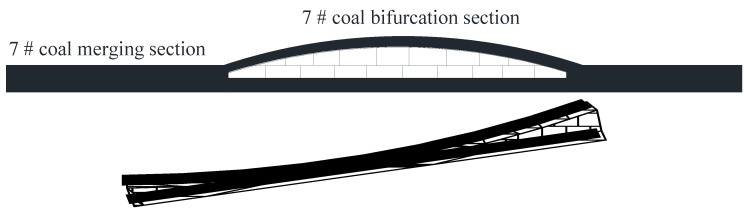
Schematic diagram of the bifurcation and merge of 7_1_ and 7_2_ coal in the Xutuan Coal Mine.

Indeed, the main mining coal seams of Xutuan Coal Mine, the 7_1_ coal seam and the 7_2_ coal seam, generally exhibit the relationship of bifurcation in the superficial parts and merger in the deep regions, as displayed in Figure 7. In this case, there is an extensive variation between the spacing of the two coal layers, and the morphological characteristics of bifurcation and merging are extremely irregular. Occasionally, the conditions of bifurcation and merger will appear in the two ends and center sections of a working face, respectively. Therefore, the thickness of the coal seam varies tremendously, which makes the selection of the stoping technology exceedingly difficult.

The basic roof of 7_1_ coal is fine sandstone with a fine grain structure, mainly quartz minerals. The direct roof of 7_1_ coal is gray or dark gray mudstone. 7_1_ coal is observed to be black and lumpy. The direct floor of 7_1_ coal (and the direct roof of 7_2_ coal) is gray or dark gray mudstone, containing fossil plant roots. The coal of 7_2_ is explored to be black and massive with endogenous fracture development, which generally contains a layer of gangue in the coal seam. The direct floor of 7_2_ coal is gray or dark gray mudstone, and a coal streak is developed in the lower part. The basic floor of 7_2_ coal is fine sandstone with gentle wavy horizontal bedding, primarily composed of quartz minerals.

Additionally, the average thicknesses of 7_1_ and 7_2_ coal refer to 1.9 m and 2.5 m, respectively. For the combined area of the coal seams 7_1_ and 7_2_, that is, the rock strata with the thickness of gangue, which is less than 1 m, the coal mining technology of full-seam mining is adopted. However, in terms of the area where the gangue is thick, full-seam mining is not suitable as a consequence of the support failure, which needs to be mined by layers. In this case, the roof of the 7_2_ coal seam is abnormally broken due to the impact of mining the 7_1_ coal seam on the floor. During the early stage of stoping 7_2_14 fully mechanized mining faces on site are affected by the mining of 7_1_ coal seams and the erosion of water, and the strength and bearing capacity of the rock mass of the roof of 7_2_ coal seams are decreased, which will lead to a broken roof and poor integrity of the working face through the process of stoping. Meanwhile, the high fraction of coal wall caving and serious roof collapse of the end face during the mining will arise, and finally, the working face is obliged to be closed in advance, which has drastically influenced the normal replacement of mines. Therefore, how to ensure the safe and efficient mining of the underlying layered 7_2_ coal in the case of slicing mining has become the major problem that the mine is facing. In this study, taking the 7_1_18 goaf of Xutuan Coal Mine as the engineering background, the influence of 7_1_ coal mining on the stability of the floor surrounding the rock system is analyzed by means of the synergetic theory of information entropy based on the failure approach index, and the grouting reinforcement range of the local damaged area of the underlying 7_2_ coal seam roof is determined, providing a guarantee for the safe and efficient mining of 7_2_ coal seam.

**Figure 8:**
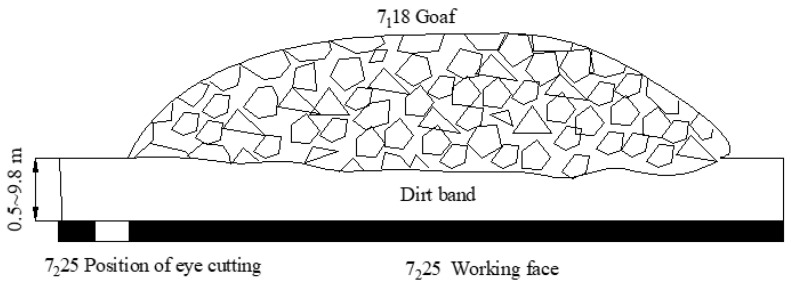
The spatial position relationship between the 7_1_18 goaf and the 7_2_25 working face.

Furthermore, the 7_2_ coal seam belongs to the lower Shihezi Formation of the lower Permian System with a thickness of 1.7 m–3.7 m and an average thickness of 2.5 m, which reflects the stable occurrence and has a spacing of 0.8 m to 9.3 m from the upper 7_1_ coal seam. The spatial relationship between the 7_1_18 goaf and the 7_2_25 working face discussed in this study is depicted in Figure 8. The elevation of the 7_1_18 goaf is −447.9 m~−480.6 m, and the ground elevation is +25.0 m.

**Figure 9:**
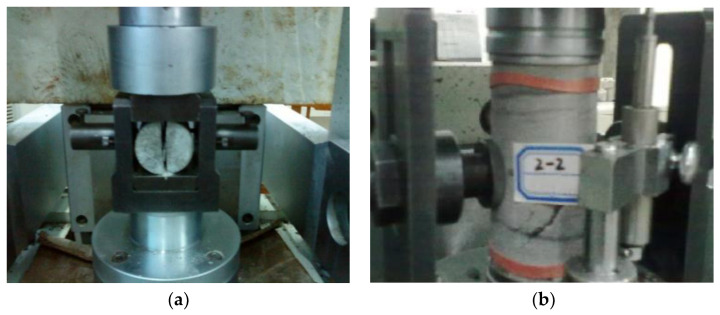
Mechanical tests: (**a**) Brazilian splitting test; and (**b**) uniaxial compression test.

To illustrate, the coal and rock sampling sites are distributed on the roof of the air roadway in the 7_2_25 working face, the falling gangue in the goaf, and the 7_1_ working face. The gangue was cored, sliced, and polished into standard cylindrical specimens through the necessary machining procedures, and the mechanical tests, such as the compressive and tensile strengths of coal and rock, were carried out by the RMT rock mechanics test system, as displayed in Figure 9. Hence, the physical and mechanical parameters of coal and rock have been obtained, as listed in Table 2.

### 4.2. Stability Analysis of the Floor Surrounding Rock System of 7_1_ Coal Seam

In light of the aforementioned numerical limit analysis method of plastic shear strain, the ultimate plastic shear strain value of mudstone in the floor of No. 7_1_ coal seam in Xutuan Coal Mine is determined to be 0.14‰, which provides a crucial criterion for the calculation of information entropy based on the failure approach index. According to the literature [30], the roof pressure step distance of No. 7_1_ coal seam mining in Xutuan Coal Mine is within the range of 8–12 m, so the model excavation range equals 15 m. Simultaneously, considering that the 7_1_18 working face is affected by a periodic weighting, the synergetic theory of information entropy based on the failure approach index is adopted to analyze the stability of the floor surrounding the rock structure after stoping on the 7_1_18 working face.

With the stoping of the working face 7_1_18, the values of the failure approach index of the floor surrounding rock unit below the goafs are extracted 1 m per advance, and the information entropy based on the failure approach index of the floor surrounding rock system is calculated by Equation (20). The sequence data is listed in Table 3.

In virtue of the sequence data of the original information entropy based on the failure approach index in Table 3, *a* = 0.1887 and *b* = 0.00023744 are calculated in accordance with Equations (34)–(36). Afterwards, $l^{\prime }$ = 15.6922 is computed in relation to Equation (40). In this way, the safety factor of the floor surrounding the rock structure equals 1.0461. Therefore, the floor surrounding the rock system of the 7_1_ coal seam proves to be firm.

**Figure 10:**
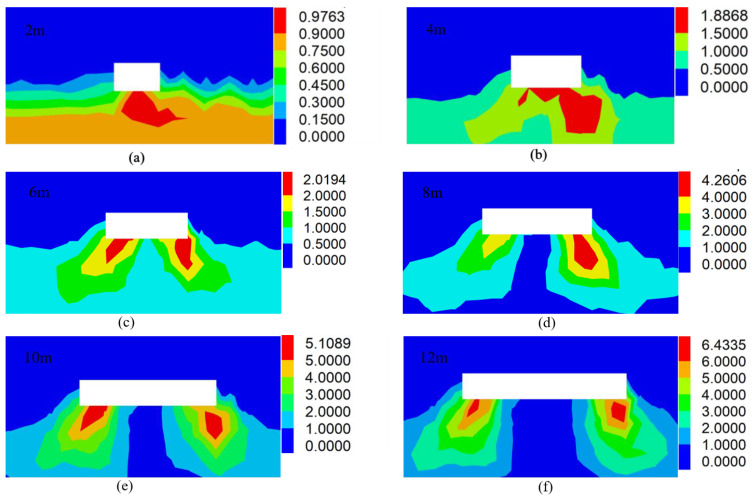

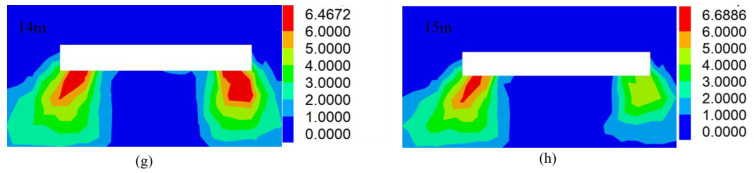
Failure cloud map from the local area of surrounding rock on the 7_1_ coal seam floor among different horizontal excavation distances: (**a**) 2 m; (**b**) 4 m; (**c**) 6 m; (**d**) 8 m; (**e**) 10 m; (**f**) 12 m; (**g**) 14 m; (**h**) 15 m.

**Figure 11:**
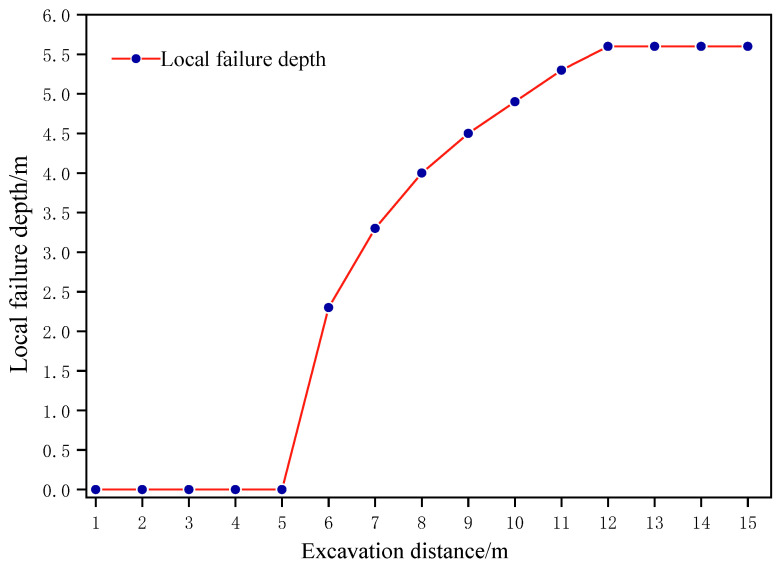
The curve of excavation distance from the 7_1_18 working face and failure depth from the local area of the floor surrounding the rock.

Although the synergetic theory of information entropy based on the failure approach index has calculated that the floor surrounding rock system of 7_1_ coal seams performs steady from a macroscopic perspective, the local area of floor surrounding rock is damaged with respect to the microscopic rock mechanics, as displayed in Figure 10. According to the partition significance of failure approach index, when FAI≥2.0, the rock and soil mass is in a state of failure [14]. When advancing 0–6 m above the 7_1_18 working face, there is no damage to the floor surrounding the rock. Nonetheless, when the working face is excavated to 6 m, the local failure of the floor surrounding the rock will emerge, and the maximum failure depth will reach 2.3 m. With the advance of the 7_1_18 coal mining work face, the abutment pressure of the stoping work face in front and behind becomes increasingly evident. When the working face is excavated to 12 m, the local failure depth of the floor surrounding the rock will reach 5.6 m. At this moment, the failure depth of the floor surrounding the rock grows to a peak of 5.6 m and tends to be stable, as exhibited in Figure 11. Consequently, it is consistent with the reference [31] that the depth of floor failure caused by the mining of 7_1_ coal is no more than 6 m. In view of the local failure area’s enormous stability influences, advanced grouting reinforcement measures should be taken to ensure the safe and efficient stoping of 7_2_ coal seams.

## 5. Conclusions

(1) Based on the strain-softened plasticity model, the general relationship between the total two-dimensional plastic shear strain and the total three-dimensional plastic shear strain has been deduced, and the numerical limit analysis method of plastic shear strain has been proposed to obtain the threshold of the ultimate plastic shear strain, which has provided the crucial criterion for the calculation of information entropy based on the failure approach index;
(2) The numerical limit analysis method was used to calculate and analyze the plastic shear strain of C25 ordinary concrete specimens, and the ultimate plastic shear strain value obtained was consistent with the result of previous studies, which verified the correctness and reliability of the numerical limit analysis method of plastic shear strain;
(3) With regard to the synergetic theory of the principle of maximum information entropy, the evolution equation of the information entropy function based on the failure approach index and excavation step of the surrounding rock system in underground mining space is established. Meanwhile, the general and solution formulae and the expression of the unstable excavation step are attained, which has presented a theoretical analysis approach for the stability analysis of the surrounding rock system;
(4) Taking the close coal seam mining project in Xutuan Coal Mine as an example, the synergetic theory of information entropy based on the failure approach index was used to analyze whether the surrounding rock system of the 7_1_ coal bottom plate with a layer thickness of 6 m is stable. Considering the periodic weighting of 7_1_ coal roofs, the local damage depth of the mining floor reaches 5.6 m. In addition, the appropriate technical measures for controlling the stability of the underlying 7_2_ coal mining roofs have been put forward to optimize the integrity of the stope roof and ensure the safety and efficiency of coal extraction.

## Figures and Tables

**Table 1:** Physical and mechanical parameters [25].

Concrete	Density *ρ*/(kg/m^3^)	Elastic Modulus *E*/(GPa)	Poisson’s Ratio *μ*	Cohesion *c*/(MPa)	Angle of Internal Friction *φ*/(°)	Tensile Strength $\sigma_{t}/(\mathrm{MPa})$
C25	2400	28	0.2	3.2	61.4	1.78

**Table 2:** Physical and mechanical parameters of each rock layer.

Lithology	Layer Thickness (m)	Density (kg/m^3^)	Elastic Modulus (GPa)	Poisson’s Ratio	Cohesion (MPa)	Internal Friction Angle (°)	Tensile Strength (MPa)
Fine sandstone	18.9	2437	27.55	0.25	3.56	35	3.6
Mudstone	1.2	2230	9.92	0.21	1.16	30	0.9
No. 7_1_ coal	1.9	1400	6.50	0.27	1.25	28	0.9
Mudstone	6.0	2230	11.03	0.22	1.92	30	1.1
No. 7_2_ coal	2.5	1400	3.80	0.21	1.46	28	0.8
Fine sandstone	15.2	2437	33.4	0.23	3.56	35	3.65

**Table 3:** Sequence data of information entropy based on the failure approach index.

Excavation Step	Information Entropy	Excavation Step	Information Entropy	Excavation Step	Information Entropy
1	3.9367	6	5.0565	11	5.3842
2	4.3509	7	5.1201	12	5.4717
3	4.7005	8	5.2080	13	5.5441
4	4.7992	9	5.2625	14	5.5578
5	5.0404	10	5.3498	15	5.6858

## Data Availability

Not applicable.
